# The combined effects of extracorporeal membrane oxygenation and renal replacement therapy on meropenem pharmacokinetics: a matched cohort study

**DOI:** 10.1186/s13054-014-0565-2

**Published:** 2014-12-12

**Authors:** Kiran Shekar, John F Fraser, Fabio Silvio Taccone, Susan Welch, Steven C Wallis, Daniel V Mullany, Jeffrey Lipman, Jason A Roberts, On behalf of the ASAP ECMO Study Investigators

**Affiliations:** Critical Care Research Group, Adult Intensive Care Services, The Prince Charles Hospital and The University of Queensland, St Lucia, Brisbane QLD 4072 Australia; Department of Intensive Care, Hôpital Erasme, Université Libre de Bruxelles, 808, Route de Lennik, 1070 Brussels, Belgium; Intensive Care Services, St Vincent’s Hospital, 683 George Street, Sydney, NSW 2000 Australia; Burns Trauma and Critical Care Research Centre, The University of Queensland, St Lucia, Brisbane, QLD 4072 Australia

## Abstract

**Introduction:**

The scope of extracorporeal membrane oxygenation (ECMO) is expanding; however, optimal drug prescription during ECMO remains a developing science. Currently, there are no clear guidelines for antibiotic dosing during ECMO. This open-label, descriptive, matched-cohort pharmacokinetics (PK) study aimed to compare the PK of meropenem in ECMO patients to critically ill patients with sepsis not receiving ECMO (controls).

**Methods:**

Eleven adult patients on ECMO (venovenous (VV) ECMO, n = 6; venoarterial (VA) ECMO, n = 5) receiving intravenous (IV) meropenem were included. Meropenem plasma concentrations were determined using validated chromatography. Population PK analysis was performed using non-linear mixed effects modelling. This data was compared with previously published meropenem PK data from 10 critically ill adult patients not on ECMO (preserved renal function (n = 5) or receiving renal replacement therapy (RRT) (n = 5). Using these data, we then performed Monte Carlo simulations (n = 1,000) to describe the effect of creatinine clearance on meropenem plasma concentrations.

**Results:**

In total, five (two VV, three VA) out of eleven ECMO patients received RRT. The other six patients (four VV, two VA) had no significant impairment in renal function. A two-compartment model adequately described the data. ECMO patients had numerically higher volume of distribution (0.45 ± 0.17 versus 0.41 ± 0.13 L/kg, *P* = 0.21) and lower clearance compared to controls (7.9 ± 5.9 versus 11.7 ± 6.5 L/h, *P* = 0.18). Variability in meropenem clearance was correlated with creatinine clearance or the presence of RRT. The observed median trough concentrations in the controls were 4.2 (0.0 to 5.7) mg/L. In ECMO patients, while trough meropenem concentrations >2 mg/L were achieved in all patients, a more aggressive target of >8 mg/L for less susceptible microorganisms was observed in only eight out of eleven patients, with five of them being on RRT.

**Conclusions:**

ECMO patients exhibit high PK variability. Decreased meropenem CL on ECMO appears to compensate for ECMO and critical illness-related increases in volume of distribution. Routine target concentrations >2 mg/L are maintained with standard dosing (1 g IV 8-hourly). However, an increase in dose may be necessary when targeting higher concentrations or in patients with elevated creatinine clearance.

## Introduction

Extracorporeal membrane oxygenation (ECMO) is being increasingly used in adult patients with acute severe cardiorespiratory failure as a supportive therapy [1,2]. Prolonged support for bridge to recovery or transplantation is now possible. While ECMO sustains life, stabilises physiology and allows time for definitive management, little is known about the independent effects of ECMO on antibiotic pharmacokinetics (PK). ECMO is thought to further complicate the PK alterations seen during critical illness [3], which appears to manifest as increased volume of distribution (Vd) and decreased clearance (CL) [4]. *Ex vivo*, animal [5,6] and clinical studies [7] are currently underway to further investigate the PK changes seen during ECMO and to develop evidence-based dosing guidelines. Simulated *ex-vivo* studies that utilised adult circuitry [8] have demonstrated significant antibiotic drug sequestration in the circuit based on physicochemical properties of individual drugs. However, the PK data on antibiotics in critically ill adult patients is limited with available studies indicating significant PK alterations [3,9,10]. This is concerning as the risks of suboptimal drug dosing (both under- and overdosing) are profound in this complex group of patients who have high infection-related mortality.

A significant number of patients receive ECMO for severe cardiac and/or respiratory failure resulting from infectious aetiologies. Patients may develop new infection during ECMO support. Studies indicate that infections occur frequently during ECMO and infections/colonisation with multi-drug-resistant organisms is not uncommon [11-13]. Gram-negative bacteria are responsible for significant proportions of these infections acquired during ECMO [11]. Meropenem is used as an empirical or targeted broad-spectrum antibiotic in this setting. It is a minimally protein-bound and hydrophilic drug that undergoes significant sequestration/degradation in *ex vivo* ECMO circuit models [8]. In this setting, one would anticipate profound alterations in meropenem PK in patients on ECMO, although to date, we are unaware of any studies to guide meropenem dosing in adults on ECMO.

This open-label, descriptive, matched-cohort PK study aimed to describe single-dose meropenem PK during ECMO using critically ill patients with sepsis and not receiving ECMO as controls.

## Materials and methods

### Participants and data collection

This study was conducted at a 650-bed university-affiliated tertiary referral hospital. The ICU is a 27-bed mixed ICU with a predominantly cardio-thoracic cohort. There is an antibiotic stewardship programme with twice weekly ward rounds by an infectious diseases physician. Infection control practices include review of all healthcare-associated bacteraemia and multiple-resistant organism screening. Ethics approval was obtained from the Prince Charles Hospital Ethics Committee, Brisbane, QLD, Australia (HREC/11/QPCH/121). Informed consent was obtained from the study participants or surrogate decision makers as applicable. The study protocol has been published and detailed methodology, inclusion and exclusion may be found elsewhere [7]. Eligible patients ≥18 years of age and receiving meropenem during their ECMO therapy were recruited. Known allergy to study drug, pregnancy, serum bilurubin concentration >150 μmol/L, ongoing massive blood transfusion requirement (>50% blood volume transfused in the previous 8 hours) and therapeutic plasma exchange in the preceding 24 hours were exclusion criteria. Data related to patient demographics, renal and hepatic function, details of ECMO and renal replacement therapy (RRT) were collected.

### Details of ECMO and RRT support

Patients received either venovenous (VV) or peripheral venoarterial (VA) ECMO as clinically indicated. The standardised ECMO circuitry comprised of Bioline tubing, Quadrox D oxygenator and a centrifugal pump (Jostra Medizintechnik AG, Hirrlingen, Germany). The prime volume was 668 mL and the circuits were freshly primed with Plasmalyte 148 (Baxter, Sydney, NSW, Australia) followed by Albumex 4% (human albumin, 40 g/L; CSL Bioplasma, Melbourne, VIC, Australia). RRT was provided as extended daily diafiltration (EDD-f) to ECMO patients using a Fresenius haemodialysis machine (4008 s ARrT plus*,* Fresenius Medical Care, Bad Homburg, Germany) that was connected to the post-oxygenator site of the ECMO circuit using Fresenius AV600S filters. The blood flow (200 to 300 mL/min) and dialysate flow rates (200 mL/min) and duration were standardised (6 to 8 hours).

Continuous venovenous haemofiltration (CVVHF) was performed in the control group RRT patients [14] using the Nephral ST500 (AN69 hollow-fibre) filter with a surface area of 2.15 m^2^. All patients were initiated on the CVVHF at least 8 hours prior to the sampling period. The ultrafiltrate rate was set between 66 and 100 mL/min, with a target blood flow rate of 250 mL/min.

### Controls

Previously published meropenem PK data were used for the historical controls (n = 10). Five patients with sepsis and no renal dysfunction receiving intermittent infusions of meropenem were included [15] from one study. The remaining five patients were the first five recruited to a PK study (n = 10) examining meropenem PK in high-volume continuous RRT [14].

### Meropenem dosing and measurements

Meropenem dosing in ECMO patients was at the discretion of the clinician, based on the clinical context and unit guidelines. The following meropenem doses were administered prior to PK sampling in the ECMO patients; 1 g intravenous (IV) bolus and 1 g IV q8h (n = 8), 1.5 g IV bolus and 1 g IV q8h (n = 2), 2 g IV bolus and 1 g IV q8h (n = 1). None of the RRT-dependent patients received an additional dose post RRT. Doses were reconstituted in 10 mL of diluent and given as IV bolus infusion in 50 mL over 30 minutes. The control patients [15] with preserved renal function (*n* = 5) were given a 1.5 g meropenem first dose (in 10 mL of water-for-injection infused by central line over 5 minutes) and then 1 g (in 10 mL of water-for-injection infused by central line over 3 minutes) every 8 hours. Controls with impaired renal function on high-volume CVVHF [14] received meropenem as 1 g (in 20 mL of water-for-injection infused by central line over 3 minutes) every 8 hours.

Blood sampling in ECMO patients was undertaken at predose, 15, 30, 45, 60, 120, 180, 360 and 480 minutes. In controls with preserved renal function [15], samples were collected at predose, 3, 5, 7, 10 15, 20, 30, 45, 60, 90, 150, 240, 360 and 480 minutes. In controls on CVVHF [14], sampling was performed at predose, and at 15, 30, 45, 60, 120, 240, and 480 minutes. All samples were immediately refrigerated at 4°C, and plasma was separated and frozen at 80°C within 24 hours of sample collection. The blood samples were centrifuged at 3,000 rpm for 10 minutes.

Meropenem analysis was conducted on a Shimadzu Prominence high-performance liquid chromatography (HPLC) system (Shimadzu Corp, Kyoto, Japan) with a Waters XBridge C18 column stationary phase (Waters Corp, Milford, MA, USA). The mobile phase was 4% acetonitrile/96% phosphate buffer 50 mM at pH 2.5 and the eluent was measured by UV at 304 nm. The internal standard for the HPLC assay was ertapenem. HPLC assays had inter- and intra-day reproducibility of 5.6% and 0.6%, respectively. The limit of quantification for meropenem was 1.0 mg/L and the coefficient of correlation for the assay was 1.000.

### Population pharmacokinetic analysis

The concentration-time data for meropenem in plasma were fitted using a non-linear mixed-effects modeling approach (NONMEM version 7.3, Globomax LLC, Hanover, MD, USA) [30]. A Digital Fortran compiler was used and the runs were executed using Wings for NONMEM [16]. Data were analysed using the first-order conditional estimation method with interaction (ADVAN3). Between-subject variability (BSV) was calculated using an exponential variability model and was assumed to follow a log-normal distribution. Residual unexplained variability (RUV) was modeled using a combined exponential and additive random error model. Visual inspection of diagnostic scatter plots and the NONMEM objective function value (OFV) were used to evaluate goodness of fit. Statistical comparison of nested models was undertaken in the NONMEM program on the basis of a *χ*2 test of the difference in OFV. A decrease in the OFV of 3.84 units (*P* <0.05) was considered statistically significant. Decreases in BSV of one of the parameters of at least 10% were also accepted for inclusion of a more complicated model. Specifically, we calculated central volume of distribution (Vc), peripheral volume of distribution (Vp), total indexed volume of distribution (Vd), inter-compartmental clearance (Q) and meropenem CL using NONMEM.

### Population pharmacokinetic model diagnostics

Visual inspection of diagnostic scatter plots and the NONMEM OFV were used to evaluate goodness of fit. Statistical comparison of nested models was undertaken in the NONMEM program using log-likelihood ratios, which are assumed to be chi-square distributed. On the basis of a *χ*2 test of the difference in OFV, a decrease in the OFV of 3.84 units (*P* <0.05) for one degree of freedom was considered statistically significant. Decreases in BSV of one of the parameters of at least 10% were also accepted for inclusion of a more complicated model.

### Population pharmacokinetic covariate screening

Covariate model building was performed in a stepwise fashion with forward inclusion and backward deletion based upon the aforementioned model selection criteria. Age, sex, weight, serum creatinine concentration, Cockroft-Gault-calculated creatinine clearance (CrCL) as well as presence of ECMO and RRT were evaluated as covariates.

### Population pharmacokinetic bootstrap

A non-parametric bootstrap method (n = 1,000) was used to study the uncertainty of the pharmacokinetic parameter estimates in the final model. From the bootstrap empirical posterior distribution, we have been able to obtain the 95% confidence interval (2.5 to 97.5% percentile) for the parameters, as described previously [17].

### Dosing simulations

We performed Monte Carlo simulations (n = 1,000) to describe the effect of five different CrCL on meropenem concentrations in a 50-year-old, 80 kg male receiving ECMO. The CrCL simulated were at 20, 50, 80, 120 and 180 mL/min. We simulated the following doses 1 g IV 8-hourly, 500 mg IV 8-hourly and 2 g IV 8-hourly. While interpreting the simulations, a trough meropenem concentration of 2 mg/L and 8 mg/L was considered optimal for treating susceptible and less susceptible pathogens, respectively [18].

### Statistical analysis

Statistical analyses were performed using the SPSS 13.0 for Windows NT software package (SPSS Inc., Chicago, IL, USA, 2004). Discrete variables were expressed as counts (percentage) and continuous variables as means ± standard deviation (SD) or median (25^th^ to 75th percentiles). Demographics and clinical differences between study groups were assessed using a chi-square, Fisher’s exact test, Student’s *t* test, or Mann-Whitney *U* test, as appropriate. A *P* <0.05 was considered to be statistically significant.

## Results

Five (two VV, three VA) out of eleven ECMO patients received RRT. The other six patients (four VV, two VA) had no significant impairment in renal and hepatic functions, based on routine biochemical parameters. The indications for ECMO included pneumonia, septic shock (n = 7); cardiogenic shock (n = 2); sickle-cell crisis (n = 1); primary graft dysfunction post lung transplant (n = 1). The median sequential organ failure assessment scores were not significantly different between the controls and ECMO patients (7 [3-15] vs. 13 [9-15,17,18], respectively, *P* = 0.14). The demographic and clinical data are summarised in Table 1. Median time to PK sampling in ECMO patients was 2 days (1 to 7).

**Table 1:** **Demography and severity of illness data**

	**Controls**		**ECMO**	
	**No RRT**	**RRT**	**No RRT**	**RRT**
	**(n = 5)**	**(n = 5)**	**(n = 6)**	**(n = 5)**
Male/Female	3/2	3/2	1/5	3/2
Age (years)	55.0 (48–61)	56 (46–66)	29 (16–46)	38 (23–56)
Total body weight (kg)	80 (75–85)	70 (60–100)	69 (60–80)	70 (70–76)
Mechanical ventilation	5/5	5/5	5/5	5/5
Type of ECMO (VA/VV)	0/0	0/0	3/3	2/3
Day 1 SOFA score	3 (3–4)	15 (14–16)	9 (7–14)	16 (13–17)
Plasma creatinine concentration (μmol/L)	73 (55–101)	na^*^	75 (44–82)	na^*^
Creatinine clearance (mL/min)	106 (98–127)	na^*^	108 (65–183)	na^*^
RRT mode	-	CVVH	-	EDD-f
Serum bilirubin (μmol/L)	9 (5–23)	93 (36–115)	23 (9–73)	58 (34–134)
Serum albumin (g/L)	22 (18–36)	26 (23–36)	31 (27–35)	24 (22–32)
Serum proteins (g/L)	56 (55–70)	62 (60–65)	49 (46–54)	44 (34–56)
Meropenem daily dose (g)	1 q 8 h	1 q 8 h	1 q 8 h	1q 8 h
Plasma C max (mg/L)	93 (74–119)	58 (52–68)	42 (27–56)	59 (50–86)
Plasma C min (mg/L)	0 (0–2)	7.5 (5–18)	4.9 (2–10)	18 (7–43)

^*^Patients RRT dependent. The biochemical indices were measured on day of pharmacokinetic sampling. Data are presented as median (IQR). ECMO, extracorporeal membrane oxygenation; RRT, renal replacement therapy; VA, venoarterial; VV, venovenous; SOFA, sequential organ failure assessment; CVVH, continuous venovenous haemofiltration; EDD-f, extended daily diafiltration.

### Meropenem concentrations and PK parameters in controls and ECMO patients

The median observed peak concentrations (C_max_) and trough meropenem concentrations (C_min_) in controls were 65.4 (58.7 to 74.4) mg/L and 4.2 (0.0 to 5.7) mg/L respectively. The ECMO group achieved a median C_max_ of 55.3 (37.8 to 60.4) mg/L and a C_min_ of 7.2 (4.0 to 17.2) mg/L; 10 out of 11 ECMO patients maintained a C_min_ >2 mg/L between doses. ECMO patients had a numerically higher, but non-statistically significant volume of distribution (0.45 ± 0.17 vs. 0.41 ± 0.13 L/kg, *P* = 0.21) and lower clearance compared to controls (7.9 ± 5.9 vs. 11.7 ± 6.5 L/h, *P* = 0.18). In ECMO patients, while trough meropenem concentrations of >2 mg/L were achieved in all patients, a more aggressive strategy of >8 mg/L targeting less susceptible microorganisms was maintained only in eight out of eleven patients, with five of them being on RRT.

### Pharmacokinetic model building

**Figure 1:**
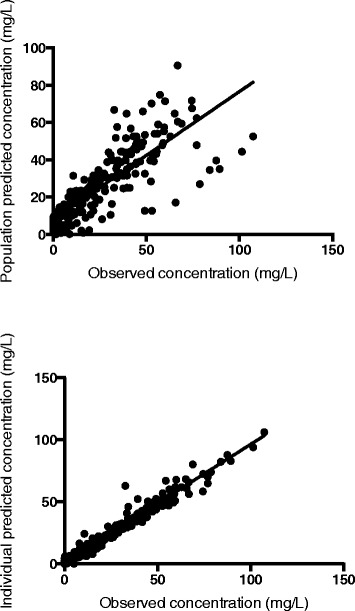
**Goodness-of-fit plots for the final covariate meropenem pharmacokinetic model**. The top panel presents the population predicted concentrations versus the observed concentrations. The lower panel presents the individual predicted concentrations versus the observed concentrations. For both graphs, the dashed line represents the lines of best fit that have acceptable correlations (r^2^ = 0.96, *P* <0.0001 for population predicted concentrations and r^2^ = 0.64, *P* <0.0001, for the individual predicted concentrations using linear regression).

The time course of plasma meropenem concentrations was best described by a two-compartment linear model with combined residual error and BSV on Vc, Vp and CL. This model included zero order input of drug into the central compartment. The mean parameter estimates from the final covariate model as well as the 95% confidence intervals from all bootstrap runs are shown in Table 2. The goodness-of-fit plots are shown in Figure 1.

**Table 2:** **Mean parameter estimates and bootstrap mean (95% confidence interval) estimates for the final covariate model**

**Parameter**	**Model**	**Bootstrap**		
	Mean	Mean	95% confidence interval	
			2.5%	97.5%
Fixed effects				
CL (L/h)	5.1	5.4	3.7	7.4
Vc (L)	18.7	18.2	13.0	21.0
Vp (L)	13.2	13.6	11.3	15.9
Q (L/h)	21.0	24.2	12.8	37.0
CL_CRCL_	1.89	1.85	0.99	2.65
Random effects BSV (% CV)				
CL (L/h)	51.6	52.2	37.9	66.6
Vc (L)	45.8	48.4	32.1	69.9
Vp (L)	28.7	16.2	0.2	42.1
Random error				
RUV (% CV)	13.7	13.3	10.1	17.6
RUV (SD, mg/L)	2.3	1.87	1.02	2.70

CL, clearance; Vc, volume of distribution of the central compartment; Vp, volume of distribution of the peripheral compartment; Q, inter-compartmental clearance; CL_CRCL_, the fractional effect of CrCL on CL for patients not receiving RRT; BSV, between-subject variability; RUV, residual unexplained variability.

After screening all relevant biologically plausible covariates, the following covariates were included in the final model. RRT was included for CL with Cockroft-Gault CrCL for CL in patients not receiving RRT. When each covariate was sequentially added to the parameters, the OFV reduced statistically significantly (*P* <0.05) and the goodness-of-fit plots improved. Inclusion of ECMO as a covariate on any parameter did not improve the goodness of fit of the model nor was it statistically significant. The final covariate model for the two-compartment meropenem model was represented by the following equation:

$$ \mathrm{TVCL} = {\theta_1}^{.}\left({\mathrm{CL}}_{\mathrm{RRT}}\right) + {\theta}_1.\ \left({\mathrm{CL}}_{\mathrm{NORRT}}*\mathrm{CrCL}\right) $$

Where TVCL is the typical value of meropenem clearance where CL_RRT_ is 0 for patients not receiving RRT and CL_NORRT_ is 0 for patients receiving RRT. CrCL is Cockroft-Gault-calculated creatinine clearance and θ_1_ is the typical population value for clearance.

### Dosing simulations

**Figure 2:**
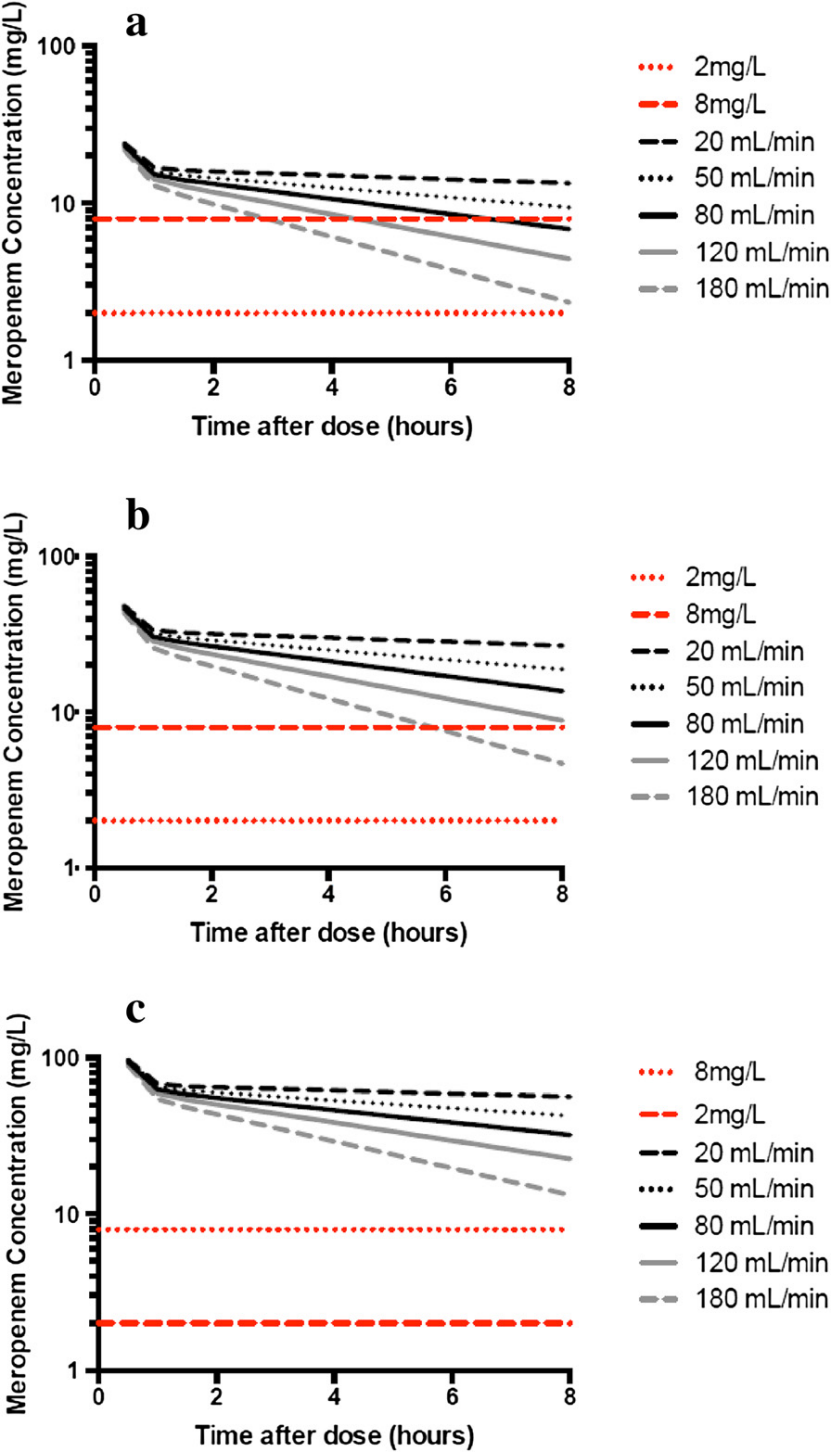
**Simulated mean meropenem logarithmic concentrations in a critically ill patient on ECMO with CrCL of 20, 50, 80, 120 and 180 mL/min for (a) 500 mg IV 8-hourly, (b) 1 g IV 8-hourly and (c) 2 g IV 8-hourly.** CrCL, creatinine clearance; ECMO, extracorporeal membrane oxygenation.

Figure 2 (a-c) shows the mean concentration-time curves for the 1,000 simulated patients for each dose and CrCL and highlights the importance of CrCL in meropenem dosing. Table 3 reports the mean and 10^th^ percentile trough concentrations for the simulated regimens. This table demonstrates the wide PK variability present in the studied patients as evidenced by the profound difference in the values described. From this data, patients with the following CrCL should receive the corresponding doses to ensure 90% of patients maintain concentrations above 2 mg/L throughout the entire dosing interval, 20 to 50 mL/min - 500 mg 8-hourly, 80 to 180 mL/min - 1 g 8-hourly, >180 mL/min - 2 g 8-hourly.

**Table 3:** **The effect of changing creatinine clearance on mean (50** ^**th**^ **percentile) and 10** ^**th**^ **percentile trough concentrations from the simulated ECMO patients (n = 1,000) receiving various meropenem doses**

			**Dose**	
**Creatinine clearance**	**Concentration percentile**	**500 mg 8-hrly**	**1 g 8-hrly**	**2 g 8-hrly**
20	50^th^	18.9	26.4	76.4
	10^th^	3.6	16.2	14.6
50	50^th^	14.5	19.7	58.8
	10^th^	2.6	9.8	10.5
80	50^th^	10.0	14.8	39.7
	10^th^	1.3	4.8	5.1
120	50^th^	7.6	11.1	30.0
	10^th^	0.7	2.5	2.7
180	50^th^	5.6	7.9	21.7
	10^th^	0.4	0.7	0.9

These simulations assume that no significant accumulation of meropenem occurred in study population. ECMO, extracorporeal membrane oxygenation.

## Discussion

This study provides preliminary evidence that standard meropenem dosing (1 g IV 8-hourly) as an intermittent bolus infusion in ECMO patients is likely to result in drug concentrations sufficient to treat highly susceptible Gram-negative pathogens. Conventional-dose meropenem should achieve a time over minimal inhibitory concentration (T_>MIC_) of 100%, assuming a minimum inhibitory concentration (MIC) of 2 mg/L (the European Committee on Antimicrobial Susceptibility Testing (EUCAST) for *Pseudomonas aeruginosa*). However, when treating less susceptible *P. aeruginosa* (MIC_90_ 8 mg/L) and *Acinetobacter* species (MIC_90_ 16 mg/L) higher meropenem doses would have to be considered especially in patients with elevated CrCL. Given that, patients on ECMO have decreased CL in most cases [3], standard dosing is likely to achieve target plasma concentrations in most patients. This is important considering the potential clinical and bacteriological benefits of maintaining 100% T_>MIC_ in critically ill patients [19].

This study uses meropenem plasma concentration data from four different patient populations to perform robust dosing simulations and to provide preliminary insights into the incremental effects of critical illness, ECMO and RRT on meropenem PK. The plasma concentrations observed in ECMO patients reflect a balance between the independent alterations in Vd and CL that occur in the presence of critical illness [20], organ failures and ECMO [3]. Interestingly in this study, routinely targeted meropenem plasma concentrations (>2 mg/L) were maintained with standard dosing, both in ECMO patients on RRT and those with preserved renal function. However, plasma meropenem concentrations were significantly higher in the RRT group when compared to patients with preserved renal function. This is important as standard dose adjustments for renal impairment (for example IV 500 mg 8-hourly or 1 g 12-hourly) in these patients receiving RRT may potentially result in under dosing. Equally, use of higher than standard doses may precipitate the risk of toxicity. Therapeutic drug monitoring where available may further improve the safety and efficacy of meropenem dosing during ECMO [4,21].

ECMO patients demonstrated reduced meropenem CL and an increased Vd when compared with controls, but these changes were not statistically significant. This trend is consistent with the PK changes expected during ECMO based on available literature [3]. An increase in Vd resulting from critical illness [20] and sequestration in the ECMO circuit [8] can significantly affect plasma concentrations probably of meropenem, a hydrophilic with limited protein binding and predominant renal CL. Equally, AKI is common in patients on ECMO, with incidence as high as 70% to 85% in single-centre studies [22]. The Extracorporeal Life Support Organisation (ELSO) Registry data [23] suggests that up to 46% of patients on VV ECMO and 44% on VA ECMO may require some form of RRT during the ECMO run. There is significant variability in mode of RRT used in ECMO patients and this may appear to limit the generalisability of our results. This to an extent has been overcome with our dosing simulations that account for a range (20 to 180 mL/min) of net CrCL (native and RRT) achieved in ECMO patients However, dosing may not be entirely based on CL achieved during RRT and possible residual renal CL or extra renal CL may have to be considered. This is of high relevance during ECMO as meropenem can undergo significant degradation/sequestration during their transit through the ECMO circuit. Although upregulated non-renal elimination is possible for ciprofloxacin in renally impaired patients [24], there is no data to support this in the case of meropenem.

In this study the estimated median meropenem CL seen in controls on CVVHF was 3.5 (3 to 4) L/h. There is no reliable data on meropenem CL during EDD-f even in non-ECMO patients and it is highly likely that this will be greater than seen with high-volume CVVHF. Despite RRT partially compensating for decreased drug CL in patients with ECMO and acute kidney injury (AKI), they maintained significantly higher meropenem concentrations during the entire dosing interval with standard dosing when compared with patients without RRT and the controls. Our simulations confirm that a meropenem dose of 500 mg - 1 g 8-hourly will provide a plasma concentration >2 mg/mL in 90% of the patients with CrCL ranging from 20 to 180 mL/min. Given the relatively wide therapeutic index of meropenem, highly variable CrCL between ECMO patients based on modality and intensity of RRT used, loss in the ECMO circuit and preponderance of less susceptible organisms in this population especially with prolonged ECMO support, a dose of 1 g 8-hourly may be considered appropriate till more PK data becomes available. Meropenem accumulation and under dosing are still potential concerns in patients with extremes of CL and these high-risk groups need to be specifically addressed in future PK studies in this population.

The findings of this study contradict the available sparse data pertaining to meropenem PK during ECMO. To our knowledge, there are no previously published PK studies in neonatal or adult patients on ECMO. A recent case report [9] indicated heightened meropenem CL (20.8 L/h) and Vd (0.56 L/kg) during ECMO and RRT and a high-dose meropenem infusion was utilised to maintain optimal concentrations. However, the CL for meropenem in the current study was significantly lower (7.3 ± 5.6 L/h) and the Vd was highly comparable (0.53 ± 0.17 L/kg). However, it should be noted that meropenem is unstable at 37°C and ongoing exteriorization of blood during ECMO may lead to a degree of spontaneous degradation, which can be erroneously interpreted as increased CL. While it is challenging to arrive at any strong conclusions based on these data, it appears that eventual success of meropenem regimens during ECMO may rely more on the CL that occurs in an individual patient.

There is emerging data to suggest that the commonly used dose of meropenem (1 g 8-hourly) may be sufficient to treat an unselected population of septic critically ill patients not receiving ECMO or RRT [25]. In this setting, the risk of under dosing with 1 g 8-hourly dosing in critically ill patients on ECMO and with preserved renal function appears small and augmented renal clearance [26] has not yet been described in this population. However, patients on peripheral VA ECMO in whom oxygenated blood is being returned into iliac artery or distal aorta may experience very high non-pulsatile renal blood flows and whether this result in heightened CL in patients with preserved renal function needs further evaluation.

The current study has limitations. Characterizing altered PK in patients receiving RRT while on ECMO can be complex. Variability in the techniques used for RRT and ECMO is a significant limitation in the generalisability of our results. Future studies should further investigate the effects of type and intensity of RRT chosen on meropenem PK during ECMO. Despite the ECMO population being small and heterogeneous, our model could accurately predict drug concentrations in ECMO patients and controls and discriminated well for RRT. This study does not address the pharmacodynamic aspects of meropenem therapy and no meaningful clinical outcome measures can be generated from the small sample. The non-ECMO patients selected were considered to be optimal controls for patients on ECMO who exhibited systemic inflammatory syndrome with or without clear evidence of infection. Systemic inflammation is known to significantly affect volume of distribution of the hydrophilic and renally excreted drugs such as meropenem [20]. Hepatic and renal function, however, were not matched as a clear separation between controls and ECMO patients who had preserved renal function and ECMO patients who were dialysis dependent, desirable in the context of this study, which sought to highlight the influence of inflammation and illness, ECMO and RRT.

## Conclusions

In patients receiving meropenem on ECMO, standard dosing (1 g 8-hourly) should achieve routinely targeted plasma concentrations. However, an increase in dose may be necessary when targeting higher plasma concentrations (>4x MIC and 100% T_>MIC_) and or in patients with elevated creatinine clearance. Future PK studies should validate these findings especially in ECMO patients with extremes of CrCL (<20 or >150 mL/min). Therapeutic drug monitoring where possible is recommended until robust dosing guidelines become available.

## Key messages

- ECMO patients exhibit high PK variability.
- Standard meropenem dosing (1 g IV 8-hourly) during ECMO achieved usual target trough concentrations of >2 mg/L both in patients with preserved renal function and in those on RRT.
- Standard meropenem dosing (1 g IV 8-hourly) during ECMO may not achieve higher target MICs (>8 mg/L), especially in patients with preserved renal function.
- Clinicians need to consider the presence of ECMO, renal function or RRT and microbiological characteristics when choosing doses for patients
- Therapeutic drug monitoring (TDM) is recommended where possible

## Abbreviations

AKI: acute kidney injury
BSV: between-subject variability
CL: clearance
CrCL: creatinine clearance
CVVHF: continuous venovenous haemofiltration
ECMO: extracorporeal membrane oxygenation
EDD-f: extended daily diafiltration
HPLC: high-performance liquid chromatography
IV: intravenous
MIC: minimum inhibitory concentration
OFV: objective function value
PK: pharmacokinetics
Q: inter-compartmental clearance
RRT: renal replacement therapy
RUV: residual unexplained variability
TDM: therapeutic drug monitoring
VA: venoarterial
Vc: central volume of distribution
Vd: volume of distribution
Vp: peripheral volume of distribution
VV: venovenous
